# Menopausal transition in multiple sclerosis: relationship with disease activity and brain volume measurements

**DOI:** 10.3389/fneur.2023.1251667

**Published:** 2023-08-03

**Authors:** Lorena Lorefice, Giuseppe Fenu, Marzia Fronza, Federica Murgia, Jessica Frau, Giancarlo Coghe, Maria Antonietta Barracciu, Luigi Atzori, Stefano Angioni, Eleonora Cocco

**Affiliations:** ^1^Department of Medical Sciences and Public Health, Multiple Sclerosis Center, Binaghi Hospital, ASL Cagliari, University of Cagliari, Cagliari, Italy; ^2^Department of Neurosciences, ARNAS Brotzu, Cagliari, Italy; ^3^Department of Biomedical Sciences, University of Cagliari, Cagliari, Italy; ^4^Radiology Unit, Binaghi Hospital, ASL Cagliari, Cagliari, Italy; ^5^Division of Gynecology and Obstetrics, Department of Surgical Sciences, University of Cagliari, Cagliari, Italy

**Keywords:** aging, brain atrophy, menopause, multiple sclerosis, neurodegeneration, oestrogen deprivation

## Abstract

**Background:**

Recent evidence has shown a significant association between menopause and multiple sclerosis (MS) progression. This study investigated the possible role of menopause in influencing MS from clinical and neuroradiological perspectives. Notably, the possible association between menopause and brain atrophy has been evaluated.

**Materials and methods:**

This study included women with MS whose ages ranged from 45 to 55 years. Demographic and clinical characteristics were collected, and the reproductive phase was defined as non-menopausal or menopausal based on the final menstrual period. Thus, MS activity over the past year was reported as the annualised relapse rate (ARR), and MRI activity (defined as new T2 lesions and/or the presence of gadolinium-enhancing lesions at the last MRI assessment in comparison with the MRI performed within the previous 12 months) were compared between non-menopausal women (non-MW) and menopausal women (MW). Volume measurements of the whole brain (WB), white matter (WM), grey matter (GM), and cortical GM were estimated using the SIENAX software, and the possible relationship with menopausal status was assessed by regression analysis.

**Results:**

The study included 147 women with MS. Eighty-four (57.1%) were MW, with a mean age of 48.5 ± 4.3 years at menopause onset and a mean duration of menopause of 4.1 ± 1.1 years. When compared for ARR, MW reported a lower rate than the non-MW (ARR of 0.29 ± 0.4 vs. 0.52 ± 0.5; *p* < 0.01). MRI activity was observed in 13.1% of MW and 20.6% of non-MW (*p* = 0.03). Lower cortical GM volumes (578.1 ± 40.4 mL in MW vs. 596.9 ± 35.8 mL in non-MW; *p* < 0.01) have also been reported. Finally, multivariate analysis showed a significant association of lower ARR (*p* = 0.001) and cortical GM volume (*p* = 0.002) with menopausal status after correction for chronological age and other variables.

**Discussion:**

Menopause may be an adverse prognostic factor of MS. Our preliminary results suggest that menopause may facilitate cortical GM atrophy, probably due to a decline in the neuroprotective effects of estrogen, with negative effects on MS evolution.

## Introduction

One of the emerging topics in the field of gender medicine applied to multiple sclerosis (MS) is the issue of menopause (1), and its effects (often superimposed on those of aging) on various aspects of the disease (2). Menopause is a physiological event that marks the end of a woman’s reproductive competence (3). Characterised by irreversible interruption of menstruation, it occurs in the general population at an average age of approximately 50 years (4). Several immunologic changes have been described in postmenopausal women. These modifications, mainly driven by oestrogen deprivation, overlap with age-related changes, resulting in decreased CD4 T lymphocytes, B lymphocytes, natural killer (NK) cells cytotoxic activity, and increased proinflammatory responses, with effects on the risk of infection and autoimmunity (5). Notably, MS is characterised by great pathogenetic, clinical, and neuroradiological heterogeneity, with different disease outcomes in relation to the inflammatory and neurodegenerative mechanisms underlying the disease (6), window for therapeutic intervention (7), and type of therapeutic intervention (8). Numerous studies have shown a predominance of the disease in females of all ages, and recent studies have shown a shift in MS onset to older age, with a higher frequency of late-onset forms among women (9). For these forms, the possible effect of menopause on susceptibility to the disease should be considered, attributable to the postmenopausal proinflammatory state and deprivation of the neuroprotective effects of oestrogens and progestins (10, 11), which would act to reduce the resilience of the central nervous system (CNS) thereby facilitating the onset of MS in the presence of other predisposing factors (12). Recently, a large study has shown that women with MS have greater inflammatory activity in terms of relapse than men, up to the age of 50 years. After that, the difference disappears, and the evolution of the disability worsens, becoming more similar in both the sexes (9, 13). Therefore, the possible effects of menopausal transition on the disease characteristics should be considered. Given the high number of women with MS among the aging population, it is crucial to understand the effects of menopause and its interaction with MS. Previously, in a longitudinal cohort of women with MS who were followed for approximately 10 years during the menopausal transition, Bove et al. showed that menopause represented an inflection point in their Expanded Disability Status Scale (EDSS) changes (14). In line with these findings, a multicentre study evaluating the effect of menopause on the clinical course of MS showed a significant decrease in annualised relapse rate (ARR) 2 years after menopause compared to the previous 2 years, while disability worsened (15). Conversely, Otero-Romero S et al. showed that menopause did not modify disability trajectories in a longitudinal cohort of women with MS who were followed from disease onset, after controlling for age and disease duration (16), thereby leaving controversial aspects to be investigated. Additionally, even less explored are the effects of menopause on neuroradiological activity and brain volume measurements in women with MS, which are significantly related to long-term disability (17). With regard to the latter point, a longitudinal study has recently shown that ovarian aging, as defined by anti-Müllerian hormone (AMH) levels, is associated with greater clinical disability and grey matter loss in women with MS (18) and is independent of the chronological age and disease duration, highlighting the crucial role of sex hormones in MS disease outcomes (19). In this framework, the present study aimed to evaluate, in a cohort of women with MS aged between 45 and 55 years, the possible impact of menopausal transition on clinical activity and MRI outcomes, and its effects on the whole brain (WB), white matter (WM), grey matter (GM), and cortical grey matter (cGM) volumes.

## Methods

### Participants

Women with relapsing–remitting MS (RRMS) (20) between the ages of 45 and 55 years were recruited from the Multiple Sclerosis Centre, Binaghi Hospital, University of Cagliari. Women were classified as as menopausal (MW) or non-menopausal (non-MW). Menopause onset was defined as the final menstrual period beyond which no menses occurred for 12 months (21) in association of neurovegetative menopausal symptoms (hot flushes). Women with surgical menopause were excluded, as were women exposed to oestrogen-progestin therapy (oral contraceptives) for up to 3 years before the final menstrual period or hormonal treatment during the menopausal transition. Demographic and clinical data [disease duration, disability level assessed by the EDSS (22), and disease-modifying therapy (DMT)], were recorded for each woman. MS clinical activity was defined as the presence of clinical relapse (new symptoms or the return of old symptoms for ≥24 h in the absence of an infection or fever). Thus, the annualized relapse rate (ARR), defined as the number of confirmed relapses in the last 12 months, was estimated after evaluation of medical records. MRI activity was defined as the presence of new or enlarged T2 lesions or gadolinium-enhancing T1 lesions at the last MRI assessment compared to the MRI performed within the previous 12 months (20). Quantitative MRI evaluations were performed for each patient, and brain volume measurements were estimated at the time of the last neurological assessment. Informed consent was obtained from all the participants after obtaining approval from the local ethics committee.

### MRI acquisition

Brain volumes were measured using a 1.5 T scanner Siemens Magnetom Avanto (Siemens Medical Solutions, Erlangen, Germany). Three-dimensional magnetisation-prepared rapid gradient-echo (MPRAGE) was used to obtain 174 contiguous sagittal 3D-T1WI images with the following parameters: slice thickness = 1.3 mm, repetition time/echo time = 2400/3.6 ms, inversion time = 1,000 ms, flip angle = 8°, field of view = 24 cm, number of excitations = 1, and pixel matrix = 192 × 192. Brain volumes were measured for each participant on T1 W gradient echo images using SIENAX, a previously described cross-sectional version of the Structural Image Evaluation using Normalisation of Atrophy (SIENA) software to estimate the global brain volume normalised for head size, as well as the selective measurement of normalised WM, GM, and cortical GM volumes (23). All brain volume measurements were performed in a single session using the same MRI protocol.

### Statistical analyses

All statistical analyses were performed using SPSS for Mac version 20.0 (SPSS Inc., Chicago, IL, United States). First, a descriptive analysis was performed, reporting demographic, clinical, and MRI data as means (quantitative variables) or percentages (qualitative variables). A t-test was used to compare demographics (age), clinical data (MS duration, EDSS score, and ARR), and MRI measurements of the WB, WM, GM, and cortical GM in non-MW and MW. Similarly, analysis of variance (ANOVA) was used to compare qualitative variables (presence of MRI activity in the last year and use of high-efficacy DMTs).

Therefore, regression analyses were performed to investigate the relationship of ARR and MRI activity (entered into the models as dependent variables) with menopausal status, while controlling for other demographic and clinical variables. Similarly, the relationship between MRI measurements of WB, WM, GM, and cortical GM volumes and menopausal status was explored using regression analyses. Statistical significance (p) was set at <0.05 for all assays.

## Results

The study included 147 relapsing remitting women with MS between the ages of 45 and 55, of whom 63 (42.9%) were non-MW and 84 (57.1%) were MW, with an average age of 48.5 ± 4.3 years at menopause onset. The mean age was 46.1 ± 3.1 years in non-MW and 52.6 ± 3.2 years in MW (*p* < 0.01), with disease duration of 15.5 ± 6.3 years and 18.2 ± 8 years, respectively (*p* < 0.05). Table 1 summarises the demographic and clinical characteristics of the patients included in this study, and also indicates the DMTs. In particular, high-efficacy DMTs were reported in 23.8% of non-MW compared to 17.8% of MW (*p* < 0.05). Table 2 shows the characteristics of non-MW and MW with clinical and neuroradiological activity in the last year of the disease and presents the comparison data of the brain MRI measurements obtained by an independent *t*-test. In particular, in the last year, an ARR of 0.52 ± 0.5 in non-MW vs. 0.29 ± 0.4 in MW (*p* < 0.01) was reported, with MRI activity observed in 20.6% of non-MW vs. 13.1% of MW (*p* < 0.05). Regression analysis was performed to evaluate the factors that influenced clinical activity, as indicated by the ARR: an inverse relationship was observed between chronological age (*p* = 0.028) and menopausal status (*p* = 0.001). Analogously, an inverse relationship that tends towards significance was observed between MRI activity and chronological age (*p* = 0.064), while no relationship was reported with menopausal status (Table 3). Multivariate analysis was then performed considering WB, WM, GM, and cortical GM as dependent variables while controlling for age, disease duration, EDSS score, and menopausal status, included in the model as independent variables. A significant association between lower cortical GM volume and menopausal status (*p* = 0.002) was reported, independent of other demographic (age) and clinical variables (MS duration and EDSS) (Table 4).

**Table 1:** Demographic and clinical features of MS women categorized in relation to menopausal status.

	MS women (147) (age range: 45–55 ys)	
	Non-menopausal MS women (63)	Menopausal MS women (84)
**Age (mean ± sd) years**	46.1 ± 3.1	52.6 ± 3.2^**^
**MS duration (mean ± sd) years**	15.5 ± 6.3	18.2 ± 8.7^*^
**EDSS score**	3.3 ± 1.8	3.4 ± 2.1
**Age at Menopause onset (mean ± sd) years**	NA	48.5 ± 4.3
**Follow-up post menopause (mean ± sd) years**	NA	4.2 ± 3.5
**Use of II° line DMTs**	15 (23.8%)	15 (17.8%)^*^

^*^p value: <0.05; ^***^p value: <0.005. Chi-square and independent-samples *t*-tests were used to compare demographic and clinical variables between the two groups.

**Table 2:** Annualized Relapse Rate, MRI activity and brain volume measurements in menopausal and non-menopausal MS women.

	Non-menopausal MS women (63)	Menopausal MS women (84)
**ARR in the last year**	0.52 ± 0.5	0.29 ± 0.4^**^
**MRI activity in the last years**	13 (20.6%)	11 (13.1%)^*^
**Whole brain** **Mean value (mL)**	1453.1 ± 57.1	1438.6 ± 74.8
**White matter** **Mean value (mL)**	688.1 ± 36.9	684.4 ± 35.4
**Grey matter** **Mean value (mL)**	764.9 ± 43.2	754 ± 50.1
**Cortical grey matter** **Mean value (mL)**	596.9 ± 35.8	578.1 ± 40.4^**^

Chi-square and independent-samples *t*-tests were used to compare clinical and MRI variables between the two groups.

**Table 3:** Multiple regression analysis.

	Annualized Relapse Rate				MRI activity			
		95% C.I. for EXP (B)				95% C.I. for EXP (B)		
	*B*	Lower	Upper	*p*	*B*	Lower	Upper	*p*
**Age**	−0.028	−0.003	−0.052	0.026	−0.022	−0.045	0.001	0.064
**MS duration**	−0.009	0.001	−0.019	0.075	0.001	−0.009	0.010	0.889
**Menopause**	−0.443	−0.664	−0.223	0.001	0.105	−0.104	0.315	0.322

Relationship of ARR with demographic, MS features and menopausal status.

**Table 4:** Multiple regression analysis.

	Cortical Grey Matter			
		95% C.I. for EXP (B)		

	*B*	Lower	Upper	*p*
**Age**	1.265	−0.623	30.152	0.188
**MS duration**	0.220	−0.556	0.996	0.576
**EDSS**	−8.972	−12.098	−5.847	0.001
**Menopause**	−28.881	−45.906	−11.856	0.002

Relationship of cortical GM volume with demographic, MS features and menopausal status.

## Discussion

Several studies have shown that natural menopause may contribute to a more rapid decline in women with MS, resulting in a turning point in the worsening of MS (14–16). These studies evaluated the impact of menopausal transition on clinical activity and EDSS changes; however, its effects on MRI activity and neurodegenerative aspects remain poorly explored. In this context, our study aimed to evaluate the impact of menopause on the course of MS, and explore the effects on MRI inflammatory activity, which is defined as an increase in lesion burden and presence of gadolinium-enhancing lesions, and the impact on brain atrophy, a principal surrogate indicator of neurodegeneration and predictor of long-term MS outcomes. In line with the results of previous studies, a relationship between lower ARR with chronological age and menopausal status was observed. At the same time, lower MRI activity appears to be associated with increasing age but is independent of menopause. It is now known that aging affects many aspects of MS (2). On the one hand, the peripheral immune response decreases, resulting in immunosenescence and making inactive plaques predominant; on the other hand, inflammation becomes compartmentalised and thus more challenging to detect, while neurodegenerative processes become more evident (24). Therefore, it is difficult to distinguish the effects exclusively linked to aging from those of menopause, which have similar effects on many aspects of immunity, brain damage, and disease evolution. Previously, Graves et al. reported that ovarian aging, as indicated by lower AMH levels, was associated with both clinical and radiographic metrics of MS severity, as shown by the relationship with lower grey matter volume after adjustments for chronological age and disease duration (18). Similarly, our study revealed an association between lower cortical grey volume and menopausal status, independent of chronological age and duration of MS, suggesting an increased level of neurodegenerative pathological processes after this reproductive biological transition. It is known that oestrogen levels decrease with menopause (3), and in line with this decrease, their neuroprotective effects decline (25). As shown in experimental autoimmune encephalomyelitis (EAE) models, oestrogen preserves synaptic transmission and has a role in sparing neurons and synapses in the brain and myelin and axons in the spinal cord (26, 27). Oestrogen exerts neuroprotective effects through various mechanisms. First, oestrogen has a suppressive effect on neuroinflammation and strongly inhibits microglial activation. In addition, a direct neuroprotective effect on the mitochondria, with increased aerobic glycolysis, respiratory efficiency, ATP generation, Ca2+ load tolerance, and antioxidant effects, has been reported (28, 29). EAE studies with various oestrogen treatments have led to clinical disease defence, as well as protection from CNS inflammation, axonal loss, demyelination, and promotion of remyelination processes (30). Thus, the reduction in the anti-inflammatory role of oestrogen after menopause could cause inflammatory damage to axons and myelin, contributing to brain damage and the accumulation of disability. Moreover, oestrogen depletion associated with menopausal transition facilitates the propensity for cardiovascular disease (4) thereby increasing the risk of aging-related comorbidities, and the impact of these comorbidities on brain damage should be considered (31). Beyond this, the effects of menopausal transition on frailty, conceived as a marker of the depletion of the organism’s homeostatic reserves (32), and on brain resilience (33) to various types of brain chronic damages (MS related or not) remain unexplored.

The present study has several limitations. First, the effects of menopausal transition on MS evolution were evaluated by comparing groups of MW and non-MW in the same age range, but not longitudinally in the same cohort. Second, most women with MS were treated with DMTs, which may have improved the course of the disease, making it more difficult to detect the effects of the menopausal transition. However, we chose not to exclude treated patients to avoid selection bias (such as the inclusion of only benign or stable MS). Furthermore, MRI data were not available for healthy controls to determine whether the association between menopause and lower GM volume was specific to women with MS. Similarly, we did not collect data of male MS patients and controls, which would have helped distinguish the effects of menopause on clinical and MRI measurements from those of aging and andropause. Furthermore, normative values for brain volumes have never been established, but only specific cut-off values capable of distinguishing between ‘physiological’ and ‘pathological’ brain volume loss in MS patients assessed longitudinally (34). However, these values are not applicable to our study since we did not longitudinally evaluate the brain volumes.

Finally, it should be emphasised that the menopausal transition process is gradual and begins even before the final menstrual period; in addition, the duration of menopause was different in the group of WM examined, while hormonal changes, which can affect the disease’s immunity, inflammation, and neurodegenerative aspects, were not evaluated in this study (5).

## Conclusion

Menopause may represent an adverse prognostic factor for MS evolution, inducing a worsening of disability and neurodegenerative aspects of MS. Our preliminary results suggest that menopause could facilitate cortical GM atrophy, probably due to a decline in the neuroprotective effects of oestrogen. In this context, further studies are needed to evaluate the impact of menopause on disease evolution. In particular, it is crucial to define studies that consider homogeneous groups of MS women, also exposed to the same type of DMT, and studies with a longitudinal design, including healthy women in the same biological phase, to define better how menopause interacts with MS and to discriminate the clinical, neuroradiological, and immunological effects induced by aging and aging-related comorbidities (35). Additionally, the effects of sex on immunosenescence and brain resilience should be further investigated with a view to facilitate an approach increasingly focused on gender medicine.

## Data availability statement

The raw data supporting the conclusions of this article will be made available by the authors, without undue reservation.

## Ethics statement

The studies involving humans were approved by Multiple Sclerosis Centre, University of Cagliari. The studies were conducted in accordance with the local legislation and institutional requirements. The participants provided their written informed consent to participate in this study.

## Author contributions

LL conceptualized the study and wrote, reviewed, and edited the manuscript. GF, MF, JF, GC, FM, and MB were responsible for resources and data curation. LA, SA, and EC supervised the study. All authors contributed to the article and approved the submitted version.

## Conflict of interest

LL, GF, JF, GC, and EC received honoraria for consultancy or speaking from Biogen, Novartis, Sanofi, Genzyme, Serono and Teva and Almirall.

The remaining authors declare that the research was conducted in the absence of any commercial or financial relationships that could be construed as a potential conflict of interest.

## Publisher’s note

All claims expressed in this article are solely those of the authors and do not necessarily represent those of their affiliated organizations, or those of the publisher, the editors and the reviewers. Any product that may be evaluated in this article, or claim that may be made by its manufacturer, is not guaranteed or endorsed by the publisher.
